# Fruit, Vegetable and Dietary Carotenoid Intakes Explain Variation in Skin-Color in Young Caucasian Women: A Cross-Sectional Study

**DOI:** 10.3390/nu7075251

**Published:** 2015-07-15

**Authors:** Kristine Pezdirc, Melinda J. Hutchesson, Ross Whitehead, Gozde Ozakinci, David Perrett, Clare E. Collins

**Affiliations:** 1 School of Health Sciences, Faculty of Health and Medicine, Priority Research Centre for Physical Activity and Nutrition, University of Newcastle, Callaghan, NSW 2308, Australia; E-Mails: kristine.pezdirc@uon.edu.au (K.P.); melinda.hutchesson@newcastle.edu.au (M.J.H.); 2 School of Medicine, University of St Andrews, Fife, KY16 9AJ, Scotland, UK; E-Mails: rw394@st-andrews.ac.uk (R.W.); go10@st-andrews.ac.uk (G.O.); 3 School of Psychology & Neuroscience, University of St Andrews, Fife, KY16 9AJ, Scotland, UK; E-Mail: dp@st-andrews.ac.uk

**Keywords:** fruit, vegetables, skin color, skin reflectance, dietary carotenoids

## Abstract

Fruit and vegetables contain carotenoid pigments, which accumulate in human skin, contributing to its yellowness. This effect has a beneficial impact on appearance. The aim was to evaluate associations between diet (fruit, vegetable and dietary carotenoid intakes) and skin color in young women. Ninety-one Caucasian women (Median and Interquartile Range (IQR) age 22.1 (18.1–29.1) years, BMI 22.9 (18.5–31.9) kg/m^2^) were recruited from the Hunter region (Australia). Fruit, vegetable and dietary carotenoid intakes were estimated by a validated food frequency questionnaire. Skin color was measured at nine body locations (sun exposed and unexposed sites) using spectrophotometry. Multiple linear regression was used to assess the relationship between fruit and vegetable intakes and skin yellowness adjusting for known confounders. Higher combined fruit and vegetable intakes (*β* = 0.8, *p* = 0.017) were associated with higher overall skin yellowness values. Higher fruit combined fruit and vegetable intakes (*β* = 1.0, *p* = 0.004) were associated with increased unexposed skin yellowness. Combined fruit and vegetables plus dietary carotenoid intakes contribute to skin yellowness in young Caucasian women. Evaluation of interventions using improvements in appearance as an incentive for increasing fruit and vegetable consumption in young women is warranted.

## 1. Introduction

Higher fruit and vegetable intakes are associated with lower risk of excess weight gain, type 2 diabetes, cardiovascular disease, and specific cancers [1,2,3]. Despite these benefits, many young women are not consuming adequate amounts of fruit and vegetables for overall health and to prevent future chronic disease. For instance, in the United States, more than 90% of women aged 19 to 30 years do not meet recommended targets [4]. In Australia, women aged 18 to 34 years have one of the lowest overall adult intakes, with only 3.7% meeting recommendations of two servings of fruit and five servings of vegetables a day [5]. Recent studies indicate that women are ambivalent about the importance of nutrition for their health [6]. Thus, finding novel strategies to motivate increased fruit and vegetables in this group is necessary to protect against chronic diseases.

Recent evidence has shown that young women are motivated to change their health behaviors based on improving their appearance or looking good rather than health concerns [7,8], which are more important amongst older females (36–50 years old). Appearance-based interventions focusing on other health risk behaviors (smoking and sun exposure) in young adults have been successful in motivating behavior change [9,10]. Similarly, a recent appearance-based intervention displaying the effects of fruit and vegetable intake on participants own facial skin color found that this approach motivated increased consumption of fruit and vegetables [11]. Interventions that focus on appearance could be a novel way of motivating young women to improve dietary intake, including fruit and vegetable intakes.

Our recent systematic review [12] examined research evidence on the relationship between food intake and appearance. Nine observational studies found significant associations between aspects of dietary intake and appearance. Findings indicated that well-conducted large observational studies using validated methods to assess exposure and outcomes are needed to further examine these relationships and to inform hypotheses and the design of future randomized controlled trials (RCTs) [12].

Fruit and vegetables contain yellow/red carotenoid pigments that, once consumed, enter the bloodstream and are distributed to various organs, including the skin [13,14]. The majority of these fat-soluble carotenoid pigments accumulate in adipose tissues, where they can be utilized as antioxidants or released back into the circulation [15]. Carotenoid pigments also accumulate in all layers of skin, including the dermis, epidermis, and stratum corneum [13,14]. This accumulation contributes to normal skin yellowness [13,14]. Carotenoid coloration (yellowness) has been reported by young adults as appearing more healthy and attractive than melanin coloration (tanning) [16,17].

Carotenoid coloration of human skin can be measured objectively using non-invasive optical methods such as resonance Raman spectroscopy (RRS) and reflectance spectroscopy [18]. A recent review conducted by Ernakov *et al*. [18] on these two optical methods highlighted their potential as objective markers of fruit and vegetable intake. Both methods have been validated against plasma carotenoid concentrations [14,19,20] and found to be valid. RRS uses laser spectroscopy, which probes the vibrational energy levels of a molecule whilst reflectance spectroscopy involves measuring skin color using CIE L*a*b* color space values and skin spectral reflectance. Evidence from previous studies using reflectance spectroscopy has shown that changes in fruit and vegetable intake [21] and use of carotenoid dietary supplements [22] over a period of time led to changes in skin redness and yellowness. However, the studies were conducted on relatively few participants (*n* = 35−82), and did not focus on young women. Further, although this previous work utilized a validated food frequency questionnaire (FFQ) to estimate fruit and vegetable intake, it only included 10 items and was developed over 25 years ago, hence it is unlikely to reflect contemporary diets. Therefore, further research using a comprehensive, validated FFQ which assesses a larger variety of fruit and vegetables items is needed to evaluate these findings in an Australian population group where mean national fruit and vegetable intakes differ, as well as potential sun exposure.

The aim of this cross-sectional study amongst young (18–30 years) Australian women is to evaluate associations between (i) fruit and vegetable intake; (ii) dietary carotenoid intake and skin color (CIE L*a*b). Secondly, the study explored the relationships between fruit, vegetable, dietary carotenoid intakes and skin reflectance in order to verify whether carotenoid pigments are responsible for any observed changes in skin color, as changes in skin color, for instance skin yellowness, can also be affected by melanin [23].

## 2. Experimental Section

### 2.1. Study Population

This cross-sectional study was conducted at the University of Newcastle, Australia, between October 2012 and June 2013, which was predominantly in Spring, Summer and Autumn. Women aged ≥18 years (*n* = 176) were recruited using flyers posted on University noticeboards, advertisements on the University’s Facebook page, and media releases. In addition, participants were recruited from the University’s School of Nursing Research Awareness Program with students given credit towards their course for research participation. The study was approved by the Human Research Ethics Committee at the University of Newcastle (H-2012–0217). An online information statement was provided to those interested, and implied consent was assumed for those who proceeded to the online survey. Written informed consent was obtained prior to physical data collection in the laboratory.

For the current analysis, women aged 18–30 years were eligible for inclusion (*n* = 112). Young women were selected as a group potentially motivated by appearance [7,8]. Current smokers (*n* = 6) were excluded due to the impact of smoking on antioxidant status, and systemic carotenoid levels [24]. Non-Caucasian women (*n* = 9) were excluded, to reduce heterogeneity of skin lightness, as the sample was predominantly Caucasian. Those who had a fake tan (*n* = 6) at the time of the assessment were also ineligible due to non-dietary impacts on skin pigmentation which is likely to affect skin lightness (L*) and yellowness (b*) [23]. In addition, no participants were excluded for skin conditions (e.g., high bilirubin, vitiligo). The final sample consisted of 91 women who completed the online survey and had completed physical assessments.

### 2.2. Measurements

Data was from participants via an online survey followed by in-person assessments, which were conducted at the University of Newcastle by Kristine Pezdirc using standardized operating procedures and protocols for the measurements. Participants were advised before their appointment to not wear make-up to their assessment. Cleansing wipes were provided for use and time was allowed for skin to dry prior to skin spectrophotometer measures being taken. In addition they were asked to refrain from any physical activity two hours prior to their appointment.

### 2.3. Skin Color and Skin Spectral Reflectance

Skin color and spectral reflectance (excluding specular reflectance component) were measured using a CM700D spectrophotometer (Konica Minolta, Osaka, Japan) with an 8 mm diameter aperture, 2-degree observer angle and illuminant D65. The spectrophotometer was white point calibrated at each measurement session.

Skin color (CIE L*a*b values, where positive L*, a* and b* values represent lightness, redness and yellowness respectively) was recorded for each participant at nine body locations on the left-hand side of the body unless stated otherwise. The nine body locations included the forehead (glabella), left and right cheek (orbitale), shoulder (acromiale), inner arm (radiale), outer arm (medial humeral epicondyle), palm (thenar muscle), waist (iliocristale) and foot sole (planta). The measurements were repeated three times at each site and the average recorded. All researchers taking measures were trained in standardize use of the spectrophotometer (by Ross Whitehead). Body locations were selected according to anatomical landmarks using the ISAK International standards for anthropometric assessment [25]. Inter-rater reliability for the nine body locations sites was assessed between five research assistants involved in the measurements using the standardized procedure. For all body locations and between experimenters across L*a* b* values the standard deviation (SD) was 0.9. The within experimenter standard deviation (SD) across the three measurements at each location was low at 0.61 across L*a*b* values.

Overall (all nine body locations), sun exposed (left and right cheeks, forehead and outer arm) and unexposed (inner arm, shoulder, hip, palm and sole of foot) L*a*b values were calculated by averaging the three measurements at each location, these were then summed and the average of those values calculated. Skin spectral reflectance (overall body locations), which measures the percentage of light reflected by skin, was measured at each site at wavelengths between 360–740 nm (10 nm intervals). Reflectance at each wavelength was divided by summed reflectance across all measured wavelengths to adjust for overall skin lightness [22]. Spectral absorption coefficient values were obtained for a subset of common carotenoids including α-carotene, β-carotene and lycopene (excluding lutein/zeaxanthin) based on those available from Miller *et al*. [26]. These coefficients were used to examine the involvement of carotenoid pigments in any observed association between skin color and diet.

### 2.4. Fruit, Vegetable and Carotenoid Intake

Dietary intake was measured using the Australian Eating Survey 2010, a validated 120-item semi-quantitative food frequency questionnaire (FFQ) that assesses dietary intake over the previous six months. The FFQ has been tested for reliability and relative validity against three-day weighed food records and is accurate for ranking nutrient intakes in Australian adults [27]. Recently, the FFQ has been validated for fruit and vegetable intake against plasma carotenoid concentrations [28]. Significant positive correlations were found between carotenoid intakes assessed by the FFQ and plasma carotenoids for α-carotene (52%, *p* < 0.001) β-carotene (47%, *p* < 0.003) and Lutein/zeaxanthin (26%, *p* = 0.041) [28]. Analysis of the mean daily nutrient intakes (including total energy intake) were performed using the latest databases available at that time, Australian AusNut 1999 (All foods), revision 17, and AusFoods (brands) Revision 5 accessed through FoodWorks version 3.02.581 (Xyris Software, Brisbane, Queensland, Australia). A standard portion size was used for each food item, determined using standard serving sizes (for example, one slice of bread) or unpublished data purchased from the Australian Bureau of Statistics data on the latest available national nutrition data at that time.

Twenty-one questions related to the intake of vegetables and 11 to fruit. Total servings of fruit and vegetables were calculated by summing the weight of relevant food items estimated by the FFQ divided by the standard serving size dictated by the Australian Guide to Healthy Eating (fruit serving 150 g, vegetable serving 75 g) [1]. The FFQ included questions on seasonal fruit. Consumption of seasonal fruits such as peach, mango, paw-paw, pineapple, melon, grapes and berries were calculated using participants’ estimation of consumption frequency when they are in season. This was adjusted by the number of months each year the fruit is available. Fruit juice was not included as part of fruit intake.

Daily carotenoid intakes for the mostly commonly consumed in the human diet α-carotene, β-carotene, lycopene and combined lutein-zeaxanthin, were calculated from FFQ fruit and vegetable responses using the US Department of Agriculture National Cancer Institute carotenoids food composition database [29].

### 2.5. Other Measures

Self-reported data for, dietary supplement use (yes or no, an open response for type of supplements, if yes), and cigarette smoking (current, past or non-smokers) were collected.

Height was measured using a portable BSM370 stadiometer correct to 0.1 cm using the stretch stature method. Weight was measured using the Inbody720 Body Composition Analyzer (Biospace Co., Ltd., Seoul, Korea). BMI was calculated using the standard equation (weight kg/height m^2^). Two measures were collected for both weight and height and averaged at the same point and the same researcher.

### 2.6. Statistical Analysis

Data analysis was undertaken using Stata (Version 11.0; StataCorp, College Station, TX, USA). A significance level was set at *p <* 0.05. Normally distributed variables are presented as means (±SD) and skewed variables are presented as medians with IQRs. Multiple linear regression was used to investigate the association between fruit, vegetable intakes (servings per day) and dietary carotenoid intakes and overall, unexposed and sun exposed skin color redness (a*) and yellowness (b*). Variables that were not normally distributed were transformed including BMI (1/square), fruit, vegetable, combined fruit and vegetable servings, dietary β-carotene, lutein, lycopene intakes (square root) and total energy and fat intakes (log).

The models were adjusted for: age, as changes in skin color are often associated with aging [30]; skin lightness (L*), as melanin affects both skin yellowness and lightness (L*) [23]; BMI and total energy intake/day as people with higher BMI and total energy intake generally consume less fruit and vegetables [31] and this is likely to be associated with lower skin yellowness [32]; supplementation use as a potential source of β-carotene or other antioxidants [22] and total fat intake (g) as carotenoids are fat soluble and bioavailability is affected by dietary fat [33,34]. As fruit and vegetable intakes are related to each other and impact on skin color the model has also been adjusted for either fruit or vegetable intake depending on the exposure variable in the analysis. Similarly dietary carotenoid intakes have been adjusted by the sum of the other carotenoids.

**Figure 1:**
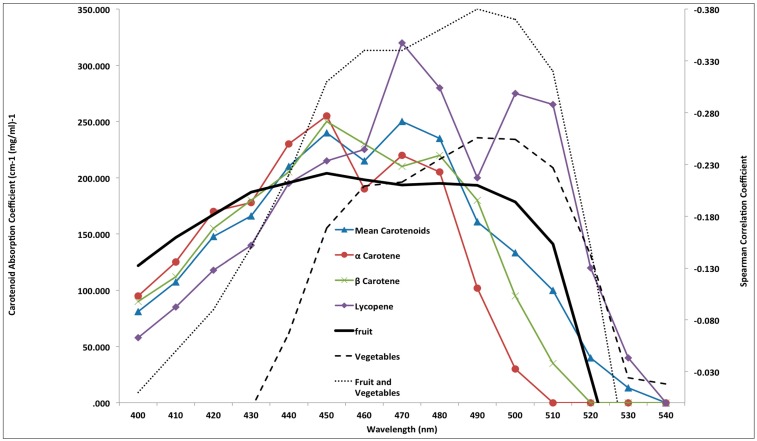
Spearman correlation coefficients between skin reflectance values and fruit, vegetable and combined fruit and vegetable consumption (servings per day) plotted with absorption spectra of common carotenoids.

Following and extending the methods used by Stephen *et al*. [22] and Whitehead *et al*. [21], partial correlations were used to investigate the relationships between fruit, vegetable or combined fruit and vegetable intake and overall skin reflectance at 10 nm intervals between wavelengths 400–540 nm (controlling for, age, fruit/vegetable servings, other carotenoids, skin lightness (L*), BMI, total daily energy, fat intake and supplement use). This was repeated for dietary carotenoid intake (α-carotene, β-carotene, lycopene and lutein). Spearman correlations were then used to assess whether the strength of these partial correlation values per wavelength was associated with the absorption spectra of common dietary carotenoids (see Figure 1). Carotenoids characteristically absorb light in the 400–540 nm region of the spectrum and reflect back longer (yellow) wavelengths, hence we expect to see negative correlations between spectral reflectance at these wavelengths and estimates of dietary carotenoid intake.

## 3. Results

### 3.1. Study Population

Of the ninety-one women included in the analyses, 86 had measurements taken on all nine sites, with the remaining five missing the hip measurement (due to the type of clothing worn during assessment). Characteristics of the sample are summarized in Table 1. The median (Interquartile Range (IQR)) age was 22.1 (19.8–26.1) years; BMI 22.9 (20.9–24.9) kg/m^2^ and percent body fat 27.9 (23.7–35.9). Thirty eight percent (*n* = 35) were taking dietary supplements with 14.3% of these participants (*n* = 5) taking supplements containing carotenoids and 51.4% taking a multivitamin that contained various antioxidants including vitamin A, C, E and zinc. Other supplements included fish oil (*n* = 5), iron (*n* = 4) and vitamin B complex (*n* = 5). Daily fruit, vegetables, dietary carotenoid, total daily energy intakes and fat intakes are summarized in Table 1. Median (IQR) fruit and vegetable intakes were 1.8 (1.0–2.7) and 3.8 (2.7–5.2) servings/day respectively, with β-carotene the most common dietary carotenoid, 6872.4 μg/day (4462.6–8918.6) (Table 1). Only 16.5% (*n* = 15) of this sample of women met current Australian recommendations of consuming at least two servings of fruit and five servings of vegetables a day [1]. Combined fruit and vegetable intake (servings) was significantly lower (*p* = 0.03) for women (*n* = 20) who were overweight or obese. Green colored vegetables and other fruits were those that were consumed in the greatest amounts with Median (IQR) intakes of 1.3 (0.8–1.7) and 0.8 (0.5–1.2) servings/day. Whilst some of these fruit and vegetables may contain large amounts of a dominant carotenoid, most contain a number of different carotenoids.

### 3.2. Associations between Fruit/Vegetable/Dietary Carotenoid Intake and Skin Color

Higher daily fruit, vegetable and combined fruit and vegetable intakes were associated with increased overall, unexposed and exposed skin redness and yellowness values except for vegetable intake and skin a* (Table 2). After adjusting for age, fruit/vegetable servings, skin lightness (L*), BMI, total daily energy, fat intake and supplement use, the associations remained significant for combined fruit and vegetable intakes (*β* = 1.0, *p* *=* 0.004), and unexposed skin yellowness values; and for combined fruit and vegetable intakes (*β* = 0.3, *p* *=* 0.049) and unexposed skin redness values. Combined fruit and vegetable intake (*β* = 0.8, *p* = 0.017) were significantly associated with overall skin yellowness values.

For dietary carotenoid intakes, only lutein/zeaxanthin was significantly associated with overall skin yellowness (*β* = 0.03, *p* = 0.010) values after adjustment for confounders (refer to supplementary data, table A1). Intakes of lutein/zeaxanthin (*β* = 0.03, *p* = 0.042) and lycopene (*β* = −0.25, *p* = 0.023) were significantly associated with unexposed skin yellowness values.

**Table 1:** Characteristics of women (*n* = 91) participating in a cross-sectional evaluation of fruit, vegetable and dietary carotenoid intake and skin color and reflectance.

Characteristic	
Age (years) median (IQR)	22.1 (19.8–26.1)
Taking Supplements Yes *n* (%)	35 (38.5)
Weight (kg) median (IQR)	62.7 (56.3–69.1)
BMI (kg/m^2^) median (IQR)	22.9 (20.9–24.9)
Fruit intake (servings/day ) median (IQR)	1.8 (1.0–2.7)
Citrus fruit ^a^ (servings/day) median (IQR)	0.1 (0.1–0.4)
Berries ^b^ (servings/day) median (IQR)	0.1 (0.1–0.3)
Core ^c^ fruits (servings/day) median (IQR)	0.7 (0.2–1.2)
Other fruits ^d^ (servings/day) median (IQR)	0.8 (0.5–1.2)
Vegetable intake (servings/day) median (IQR)	3.8 (2.7–5.2)
Orange/yellow ^e^ colored vegetables (servings/day) median (IQR)	0.8 (0.5–1.1)
Red ^f^ colored vegetables (servings/day) median (IQR)	0.6 (0.2–0.9)
Green ^g^ colored vegetables (servings/day) median (IQR)	1.3 (0.8–1.7)
White ^h^ colored vegetables (servings/day) median (IQR)	0.8 (0.4–1.3)
Total fruit and vegetable intake (servings/day) median (IQR)	5.9 (4.1–7.4)
**Dietary Carotenoids**	
α-carotene (μg/day) median (IQR)	1988.6 (1220.2–2611.6)
β-carotene (μg/day) median (IQR)	6872.4 (4462.6–8918.6)
Lutein zeaxanthin (μg/day) median (IQR)	2276.8 (1523.6–2895.1)
Lycopene (μg/day) median (IQR)	5054.8 (2975.1–7488.5)
Total energy (kilojoules/day) median (IQR)	7921 (6538–9801)
Total fats (grams/day) median (IQR)	66.9 (53.9–84.0)
Overall L* mean ± SD	65.2 ± 2.1
Overall a* mean ± SD	9.3 ± 1.2
Overall b* mean ± SD	16.3 ± 2.1
Unexposed L* mean ± SD	66.5 ± 2.2
Unexposed a* mean ± SD	7.3 ± 1.2
Unexposed b* mean ± SD	16.3 ± 2.2
Exposed L* mean ± SD	63.5± 2.3
Exposed a* mean ± SD	11.8 ±1.4
Exposed b* mean ± SD	16.3 ± 2.1

^a^ Orange, mandarin, grapefruit; ^b^ grapes, strawberries, blueberries; ^c^ apples, pears; ^d^ banana, stone fruits, mango, pineapple, and melons; ^e^ pumpkin, sweet potato, carrots and corn; ^f^ tomato and capsicum; ^g^ green beans, spinach, broccoli, peas and lettuce; ^h^ potato, cauliflower, mushrooms and onion.

**Table 2:** Multiple regression analyses between fruit and vegetable intake and skin color (CIE L*a*b*).

	Unadjusted			Adjusted ^a^		
	β-Coefficient ± SE	95% Confidence Interval	*p* value	β-Coefficient ± SE	95% Confidence Interval	*p* value
Overall Redness (a *) *n* = 86						
Fruit (servings/day)	0.8 ± 0.3	(0.3, 1.3)	0.002	0.02 ± 0.2	(−0.4, 0.4)	0.918
Vegetable (servings/day)	0.6 ± 0.3	(0.1, 1,1)	0.017	0.2 ± 0.2	(−0.1, 0.6)	0.162
Combined Fruit and vegetables (servings/day)	0.7 ± 0.2	(0.2, 1.1)	0.003	0.2 ± 0.1	(−0.1, 0.5)	0.167
Overall Yellowness (b *) *n* = 86						
Fruit (servings/day)	1.8 ± 0.4	(0.9, 2.6)	<0.001	0.9 ± 0.4	(−0.1, 1.8)	0.065
Vegetable (servings/day)	1.4 ± 0.4	(0.6, 2.3)	0.002	0.4 ± 0.4	(−0.4, 1.1)	0.297
Combined Fruit and vegetables (servings/day)	1.5 ± 0.4	(0.8, 2.2)	<0.001	0.8 ± 0.3	(0.1, 1.4)	0.017 *
Unexposed ^b^ Redness (a *) *n* = 86						
Fruit (servings/day)	0.8 ± 0.3	(0.3, 1.3)	0.005	0.2 ± 0.2	(−0.3, 0.6)	0.406
Vegetable (servings/day)	0.8 ± 0.3	(0.3, 1.3)	0.004	0.3 ± 0.2	(−0.1, 0.6)	0.135
Combined Fruit and vegetables (servings/day)	0.7 ± 0.2	(0.3, 1.2)	0.001	0.3 ± 0.2	(0.007, 0.6)	0.049 *
Unexposed Yellowness (b *) *n* = 86						
Fruit (servings/day)	1.9 ± 0.5	(1.0, 2.9)	<0.001	0.9 ± 0.5	(−0.04, 1.9)	0.060
Vegetable (servings/day)	1.8 ± 0.5	(0.9, 2.7)	<0.001	0.6 ± 0.5	(−0.2, 1.5)	0.120
Combined Fruit and vegetables (servings/day)	1.8 ± 0.4	(1.0, 2.5)	<0.001	1.0 ± 0.4	(0.3, 1.7)	0.004 *
Exposed ^c^ Redness (a *) *n* = 91						
Fruit (servings/day)	0.8 ± 0.3	(0.2, 1.4)	0.009	−0.1 ± 0.3	(−0.6, 0.4)	0.676
Vegetable (servings/day))	0.4 ± 0.3	(−0.2, 1.0)	0.147	0.2 ± 0.2	(−0.2, 0.6)	0.390
Combined Fruit and vegetables (servings/day)	0.5 ± 0.3	(0.1, 1.0)	0.029	0.1 ± 0.2	(−0.3, 0.4)	0.622
Exposed Yellowness (b *) *n* = 91						
Fruit (servings/day)	1.5 ± 0.4	(0.7, 2.4)	0.001	0.9 ± 0.5	(−0.1, 1.8)	0.080
Vegetable (servings/day)	1.1 ± 0.4	(0.2, 1.9)	0.015	0.2 ± 0.4	(−0.5, 1.0)	0.558
Combined Fruit and vegetables (servings/day)	1.2 ± 0.4	(0.5, 1.9)	0.001	0.6 ± 0.3	(−0.04, 1.3)	0.065

^a^ Adjusted for fruit/vegetable servings L*, supplementation use, BMI, total energy intake, fat intake and age; ^b^ Unexposed: Total of Inner arm, shoulder, hip, palm and sole of foot; ^c^ Exposed: Total of face (left and right cheeks, forehead) and outer arm; * Significant *p* < 0.05.

### 3.3. Relationships between Fruit, Vegetable, Dietary Carotenoid Intakes and Skin Reflectance

Table 3 and Figure 1 demonstrate that the partial correlations between fruit intake and overall skin spectral reflectance across wavelengths 400–540 nm is significantly and negatively correlated with the absorption spectra of α-carotene (*r* = −0.89, *p* < 0.001), β-carotene (*r* = −0.97, *p* < 0.001), lycopene (*r* = −0.69, *p* = 0.0047) and the mean absorption of these three common carotenoids (*r* = −0.94, *p* < 0.001). The relationship between vegetable intake and skin reflectance across wavelengths 400–540 nm was significantly negatively correlated with the absorption spectra of lycopene (*r* = −0.80, *p* = 0.0004). The relationship between combined fruit and vegetable intake and skin reflectance was significantly negatively correlated with the absorption spectra of β-carotene (*r* = −0.58, *p* = 0.0236), lycopene (*r* = −0.90, *p* < 0.001) and mean carotenoid (*r* = −0.65, *p* = 0.0086). The correlation coefficients between dietary intakes of β-carotene (*r* = −0.69 *p* = 0.0045) and lycopene (*r* = 0.82, *p* = 0.0002*)* with their respective absorption spectra were significant.

**Table 3:** Relationship between partial correlation coefficients between fruit, vegetable and dietary carotenoid intake, overall skin reflectance and absorption spectra of common carotenoids as measured by spectrophotometry.

	α-Carotene		β-Carotene		Lycopene		Mean Carotenoids	
	ρ	*p* value	ρ	*p* value	ρ	*p* value	ρ	*p* value
Fruit (servings/ day)	−0.89 *	<0.001	−0.97 *	<0.001	−0.69 *	0.0047	−0.94 *	<0.001
Vegetable (servings/ day)	−0.08	0.7641	−0.31	0.2603	−0.80 *	<0.0004	−0.40 *	0.1435
Combined Fruit and vegetables (servings/ day)	−0.37	0.1734	−0.58 *	0.0236	−0.90 *	<0.0001	−0.65 *	0.0086
α-carotene intake (μg/day)	−0.31	0.2576						
β-carotene intake (μg/day)			−0.69	0.0045				
Lycopene intake (μg/day)					0.82 *	0.0002		

* Significant *p* < 0.05. Spearman correlations were used to assess whether the strength of these partial correlation values was associated with the absorption spectra of common dietary carotenoids.

## 4. Discussion

The current study demonstrates that higher fruit and vegetable and dietary carotenoid intakes in young Australian women are associated with skin color, especially skin yellowness. In the adjusted regression models, for every additional serving of combined fruit and vegetables per day there was an increase of 0.8 units in overall skin yellowness and 1.0 units in unexposed skin yellowness. A Delta E (*Δ*E) of 1.37 to 1.55 in skin color change which is a very small difference (*Δ*E is defined as the difference between colors in the CIE L*a*b* color space) is perceived as a visible increase in the appearance of skin as healthy and attractive [21].

As anticipated, negative significant correlations were found between fruit and vegetable intakes and skin reflectance at wavelengths associated with light absorption by carotenoids. For fruit consumption, these relationships were strongest at wavelengths associated with high light absorption by common dietary carotenoids (α-carotene, β-carotene and lycopene). For vegetable intake it was only demonstrated for the absorption spectra of lycopene. In addition, both dietary lycopene and β-carotene were significantly correlated with their absorption spectra. This provides further evidence that human skin coloration is related to fruit and vegetable consumption, with this relationship strongest at the specific wavelengths associated with greater light absorption by carotenoid pigments.

Our findings support evidence from previous studies reporting that higher fruit and vegetable consumption was associated with higher skin yellowness [21,22]. Using a 63-item FFQ, 10 of which related to fruit and vegetables, Whitehead *et al.* [21] examined change in fruit and vegetable intake over a six-week period. They found a modest increase in consumption was associated with a significant increase in skin yellowness and redness in both exposed and unexposed skin. In the current cross-sectional study, after adjusting for fruit/vegetable servings, BMI, dietary supplement use, skin lightness, total energy, fat intake and age which were not accounted for (except skin lightness) in previous studies, only the relationship between combined fruit and vegetables and skin yellowness in unexposed skin remained statistically significant. This suggests that the relationship between diet and skin color is strongest at unexposed areas of the skin. This may be because sun exposed skin might be less likely to show dietary effects because carotenoids are locally expended by UV exposure [13]. These findings however need to be confirmed in larger prospective cohort studies using objective measures of carotenoid intakes such as plasma carotenoids and reflectance spectrophotometry.

This is the first study to explore the association between dietary lutein/zeaxanthin and skin color. We found significant associations between lutein/zeaxanthin intake and overall, unexposed and exposed skin yellowness after adjusting for confounders. This could possibly be because the relative bioavailability of lutein in particular, if consuming a variety of vegetables, is much greater than that of β-carotene [34]. However there was no significant association between vegetable intake and skin yellowness. In addition previous research has shown that lutein and zeaxanthin accumulate in macula of the retina rather than in the skin [20]. No data could be reported on the relationship between the absorption spectrum of lutein and diet because dietary lutein/zeaxanthin intake has been calculated together.

Unlike the findings from Whitehead *et al*. [21], the only significant relationships between combined fruit and vegetable intakes with skin redness were observed in unexposed areas. Lycopene, a pigment that is found in tomatoes and other fruits and vegetables may contribute to skin redness, just as β-carotene impacts skin yellowness [21]. Hence one possibility for the difference in findings is the differing food lists in each respective FFQ used, with the FFQ in the current study potentially failing to capture all sources of dietary lycopene, or that lycopene intakes were low, hence the lack of significant relationship between fruit and/or vegetable intake and skin redness.

Strengths of the current study include the objective measurement of skin color, which was based on standardized anthropometric sites [25]. Assessors were trained prior to the study starting and inter-reliability was tested amongst researchers and multiple measures performed on both sun exposed and unexposed sites for each participant. The main limitation is the cross-sectional design of the current study. Hence only associations between intake and skin color can be evaluated. While the relationship was adjusted for potential confounders it is possible that the residual confounding due to other factors associated with fruit and vegetable consumption could account for some of the relationship seen. Other potential limitations include the use of the FFQ to measure carotenoid intake rather than using the objective measure of plasma carotenoids. However this FFQ has been shown to provide a valid estimate of fruit and vegetable intake in comparison to plasma carotenoid concentrations [28]. The USDA database was used to calculate dietary carotenoids. This database is limited with respect to the foods that contain carotenoids although it is the most updated and reliable database available. In addition the FFQ does not include questions on how the food was prepared (e.g., with or without fat, raw or heated) as these factors highly influence the bioavailability of carotenoids [34]. The impact of the food processing method on bioavailability of carotenoids could not ascertained for all food items, hence results should be interpreted with caution as the bias could impact the relationship in either direction. Fruit juices were excluded from the analysis as the questions in the FFQ ask about the frequency of consumption of fruit based drinks (e.g., orange juice or poppers). These are considered as sweetened beverages and are categorized in the FFQ as non-core food/drinks. This could potentially have underestimated carotenoid intakes hence biasing the results. The FFQ reports intake over the previous six months and carotenoids are absorbed into the bloodstream after three to four days [34]. These limitations may explain why correlations between dietary carotenoid intakes and their respective absorption spectra were not found. Only Caucasian women were included due to very small numbers from other ethnicities being recruited, which limits the external validity to this population group only. In addition, the reported fruit and vegetable intake for this group of women was high compared to the Australian population [5], which could have been due to FFQ overestimation or that those participating were more health conscious compared to the general population. This also impacts external validity, as we cannot compare these results to women who have a lower intake of fruit and vegetables. In addition, The FFQ has been validated in the adult population for nutrient intakes [27] (which includes β-carotene) and has been validated for fruit and vegetable intake [28]. There was no data collected on physical activity and hence any potential effects of exercise on skin color cannot be controlled for. There is evidence to suggest that people who consume a healthy diet eat more fruit and vegetables and engage in physical activity [22].

### Study Implications

Evidence suggests that young women are motivated to change their behavior by appearance rather than health [7,8]. The results of the current study provide support that higher consumption of fruit and vegetables is associated with higher skin yellowness. Previous evidence has suggested that this has a beneficial effect on both perceived and actual appearance. Studies by Whitehead *et al*. [21] and Stephen *et al*. [22] have shown that individuals find the yellow coloration of skin healthier and more attractive than tanned skin [16,17]. Therefore future interventions could focus on improving young women’s skin color to improve their perceived health and appearance, to motivate them to increase their fruit and vegetable intake.

The findings of the current study suggest that further research is required to determine if spectrophotometer assessment has the potential for use as a biomarker for fruit and vegetable intake. Plasma carotenoids are a biological marker of fruit and vegetable intake, however analysis is expensive and labor intensive, whilst dermal spectrophotometry is non-invasive, easy to use, and generates instant results. Skin carotenoids measured by RRS have been validated against plasma carotenoids and indicate that lycopene, β-carotene and total carotenoids are significantly correlated with their respective plasma concentrations [19,35]. Future research should examine the relationship between repeated measurement of skin color using the spectrophotometer and long-term carotenoid status in comparison with plasma carotenoid concentrations.

## 5. Conclusions

In conclusion, the current study provides evidence that fruit, vegetable and dietary carotenoid intake has an impact on skin color, in particular skin yellowness. Further research across a range of ethnicities other than Caucasians and including males is warranted to evaluate these relationships further. Future research evaluating whether carotenoid enrichment in skin, as measured by spectrophotometry, can be detected as a result of interventions that promote greater intakes of fruit and vegetable is warranted. Further work is also required to determine whether the observed effects could be used as a tool to motivate young women to change their dietary behavior.

## Appendix

**Table A1:** Multiple regression analyses between dietary carotenoid intake and skin color (CIE L*a*b*).

	Unadjusted				Adjusted ^a^				
	β-Coefficient ± SE	95% Confidence Interval	*p* value		β-Coefficient ± SE	95% Confidence Interval		*p* value	
**Overall Redness (a *) *n* = 86**									
α-carotene intake (μg/day)	0.00 ± 0.00	(–0.00, 0.00)	0.101		0.00 ± 0.00	(–0.00, 0.00)		0.370	
β-carotene intake (μg/day)	0.02 ± 0.01	(0.00, 0.03)	0.015		–0.001 ± 0.01	(–0.02, 0.02)		0.918	
Lycopene intake (μg/day)	0.00 ± 0.01	(–0.01, 0.02)	0.665		0.00 ± 0.00	(–0.00, 0.01)		0.249	
Lutein zeaxanthin intake (μg/day)	0.02 ± 0.01	(0.01, 0.04)	0.010		0.00 ± 0.01	(–0.01, 0.14)		0.876	
**Overall Yellowness (b *) *n* = 86**								
α-carotene intake (μg/day)	0.00 ± 0.00	(–0.00, 0.00)	0.089		0.00 ± 0.00	(–0.00, 0.00)	0.173	
β-carotene intake (μg/day)	0.03 ± 0.01	(0.01, 0.05)	0.009		0.01 ± 0.02	(–0.03, 0.04)	0.741	
**Unexposed ^b^ Redness (a *) *n* = 86**								
Lycopene intake (μg/day)	–0.03 ± 0.01	(–0.05, –0.01)	0.011		–0.02 ± 0.01	(–0.04, 0.00)	0.051	
Lutein zeaxanthin intake (μg/day)	0.06 ± 0.01	(0.03, 0.09)	0.000		0.03 ± 0.15	(0.00, 0.06)	0.045	
α-carotene intake (μg/day)	0.00 ± 0.00	(–0.00, 0.00)	0.063		0.00 ± 0.00	(–0.00, 0.00)	0.611	
β-carotene intake (μg/day)	0.02 ± 0.01	(0.01, 0.03)	0.003		0.01 ± 0.01	(–0.01, 0.03)	0.342	
Lycopene intake (μg/day)	0.00 ± 0.01	(–0.01, 0.02)	0.720		0.01 ± 0.00	(–0.00, 0.01)	0.269	
Lutein zeaxanthin intake (μg/day)	0.03 ± 0.01	(0.01, 0.05)	0.000		0.01 ± 0.01	(–0.01, 0.02)	0.397	
**Unexposed Yellowness (b *) *n* = 86**								
α-carotene intake (μg/day)	0.00 ± 0.00	(0.00, 0.00)		0.025	0.00 ± 0.00	(–0.00, 0.00)	0.082	
β-carotene intake (μg/day)	0.04 ± 0.01	(0.02, 0.06)		0.001	0.02 ± 0.02	(–0.25, 0.58)	0.437	
Lycopene intake (μg/day)	–0.03 ± 0.01	(–0.05, –0.005)		0.019	–0.25 ± 0.01	(–0.05, –0.00)	0.023	
Lutein zeaxanthin intake (μg/day)	0.07 ± 0.01	(0.04, 0.10)		0.000	0.03 ± 0.02	(0.00, 0.07)	0.042	
**Exposed ^c^ Redness (a *) *n* = 91**								
α-carotene intake (μg/day)	0.00 ± 0.00	(–0.00, 0.00)		0.282	0.00 ± 0.00	(–0.00, 0.00)	0.323	
β-carotene intake (μg/day)	0.01 ± 0.00	(–0.00, 0.03)		0.144	–0.01 ± 0.01	(–0.31, 0.01)	0.274	
Lycopene intake (μg/day)	0.00 ± 0.01	(–0.01, 0.02)		0.745	0.00 ± 0.01	(–0.01, 0.01)	0.415	
Lutein zeaxanthin intake (μg/day)	0.01 ± 0.001	(–0.01, 0.03)		0.258	–0.00 ± 0.01	(–0.02, 0.01)	0.631	
**Exposed Yellowness (b *) *n* = 91**								
α-carotene intake (μg/day)	0.00 ± 0.00	(–0.00, 0.00)		0.333	0.00 ± 0.00	(–0.00, 0.00)	0.519	
β-carotene intake (μg/day)	0.02 ± 0.01	(–0.00, 0.04)		0.068	0.00 ± 0.02	(–0.04, 0.04)	0.961	
Lycopene intake (μg/day)	0.03 ± 0.01	(–0.05, –0.01)		0.012	–0.01 ± 0.01	(–0.03, 0.01)	0.279	
Lutein zeaxanthin intake (μg/day)	0.05 ± 0.01	(0.02, 0.01)		0.001	0.03 ± 0.02	(–0.01, 0.06)	0.099	

^a^ Adjusted for total other carotenoid intake, L* , supplementation use, BMI, total energy intake, fat intake and age; ^b^ Unexposed: Total of Inner arm, shoulder, hip , palm and sole of foot; ^c^ Exposed: Total of face (left and right cheeks, forehead) and outer arm.
